# Pectus excavatum in blunt chest trauma: a case report

**DOI:** 10.1186/1752-1947-7-22

**Published:** 2013-01-15

**Authors:** Emmanouil Liodakis, Eirini Liodaki, Hrayr G Basmajian, Nael Hawi, Maximilian Petri, Christian Krettek, Michael Jagodzinski

**Affiliations:** 1 Trauma Department, Hannover Medical School, Carl-Neuberg-Str 1, Hannover, 30625, Germany; 2 Department of Plastic Surgery, University Clinic of Schleswig-Holstein, Campus Lübeck, Germany; 3 Department of Orthopaedic Surgery, Loma Linda University Medical Center, Loma Linda, CA, USA

**Keywords:** Blunt cardiac rupture, Pectus excavatum, Seatbelt injury

## Abstract

**Introduction:**

Blunt cardiac rupture is an exceedingly rare injury.

**Case presentation:**

We report a case of blunt cardiac trauma in a 43-year-old Caucasian German mother with pectus excavatum who presented after a car accident in which she had been sitting in the front seat holding her two-year-old boy in her arms. The mother was awake and alert during the initial two hours after the accident but then proceeded to hemodynamically collapse. The child did not sustain any severe injuries. Intraoperatively, a combined one-cm laceration of the left atrium and right ventricle was found.

**Conclusion:**

Patients with pectus excavatum have an increased risk for cardiac rupture after blunt chest trauma because of compression between the sternum and spine. Therefore, patients with pectus excavatum and blunt chest trauma should be admitted to a Level I Trauma Center with a high degree of suspicion.

## Introduction

Blunt cardiac rupture is an exceedingly rare injury. Patients with cardiac rupture following blunt thoracic trauma rarely survive and most die at the scene of the accident or soon after in the emergency room, before the cardiac lesions are detected [1,2]. Cardiac squeezing between the sternum and spine is postulated as the most likely trauma mechanism [3,4].

Pectus excavatum accounts for 90% of congenital chest wall deformities and refers to the posterior depression of the sternum and adjacent costal cartilages. Pectus excavatum is more than a cosmetic deformity because it can cause cardiopulmonary impairment and physiologic limitations. The depressed sternum often compresses the right atrium as well as the right ventricle [5]. Rationally, the incidence of cardiac ruptures should be higher in patients with pectus excavatum.

We report a case of a woman who survived rupture of the left ventricle following blunt chest trauma after presenting hemodynamically stable and without signs of pericardial tamponade.

## Case presentation

**Figure 1:**
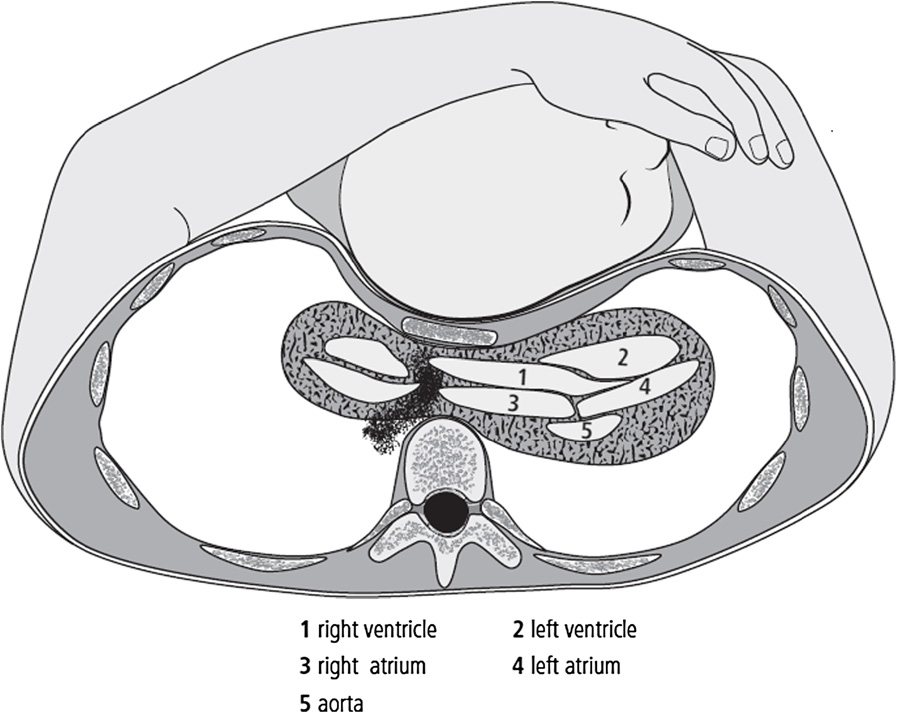
**A young mother with pectus excavatum holding her two-year-old boy in her arms during the car accident. **Caudal view.

A 43-year-old Caucasian German woman wearing a three-point seatbelt and holding her two-year-old boy in her arms in the front passenger seat of a car was involved in a head-on collision with a tree (Figure 1). When the emergency team arrived at the scene of the accident, the car cabin was slightly deformed, the driver and passenger had their seat belts on, and both airbags were not deployed. The driver had some minor injuries and was transported to another hospital; the patient and her son were transported to the emergency room at a level-1 trauma hospital secondary to complaints of thoracic pain and lower limb pain, respectively. No visible evidence of trauma including a seat belt sign was noted on the chest of the women or the child’s head.

**Figure 2:**
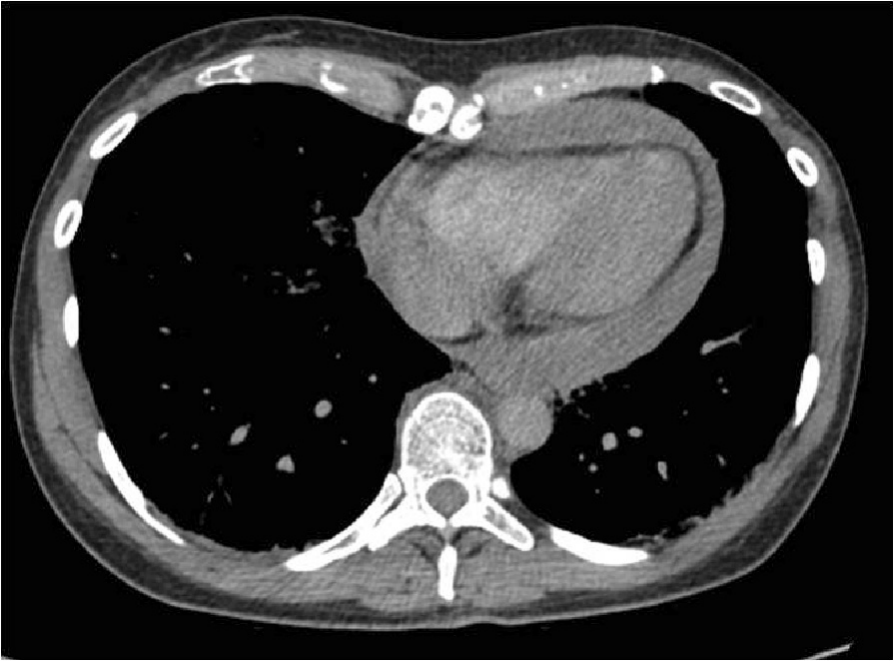
Computed tomography scan showing the three-cm pericardial effusion.

They presented to the emergency department at our trauma hospital one hour and ten minutes after the accident. On admission, the mother was alert (Glasgow Coma Scale 15) and hemodynamically stable (blood pressure 120/70mmHg; 84 pulses/minute). The usual clinical examination was conducted following Advanced Trauma Life Support principles. The trauma surgeon performing the Focused Assessment with Sonography for Trauma did not detect any free intraperitoneal fluid. The initial radiological assessment (chest, pelvic and cervical spine X-ray) showed the clinically evident pectus excavatum, multiple rib fractures and a stable, not dislocated, pelvic fracture. Fifteen minutes after admission she was transported alert and in a cardiopulmonary stable state to the computed tomography (CT) scanner. The CT scan demonstrated a zygomatic fracture, three-cm pericardial effusion (Figure 2), fractures of the 2nd and 3rd ribs, and a pelvic type C fracture.

Two hours after the trauma, the patient became hemodynamically unstable (blood pressure 70/40mmHg; 122 pulses/minute). Her hemoglobin level was 9.5g/dL (on admission 10.4g/dL) and the pH 7.27 (Additional file 1: Table S1). The trauma surgeon immediately performed a pericardiocentesis with insertion of a pigtail catheter to temporarily stabilize the patient and bridge the time until surgery. Intubation and central vein and artery catheterization followed immediately. The on-call cardiovascular surgeon began the surgery four hours after the trauma.

**Figure 3:**
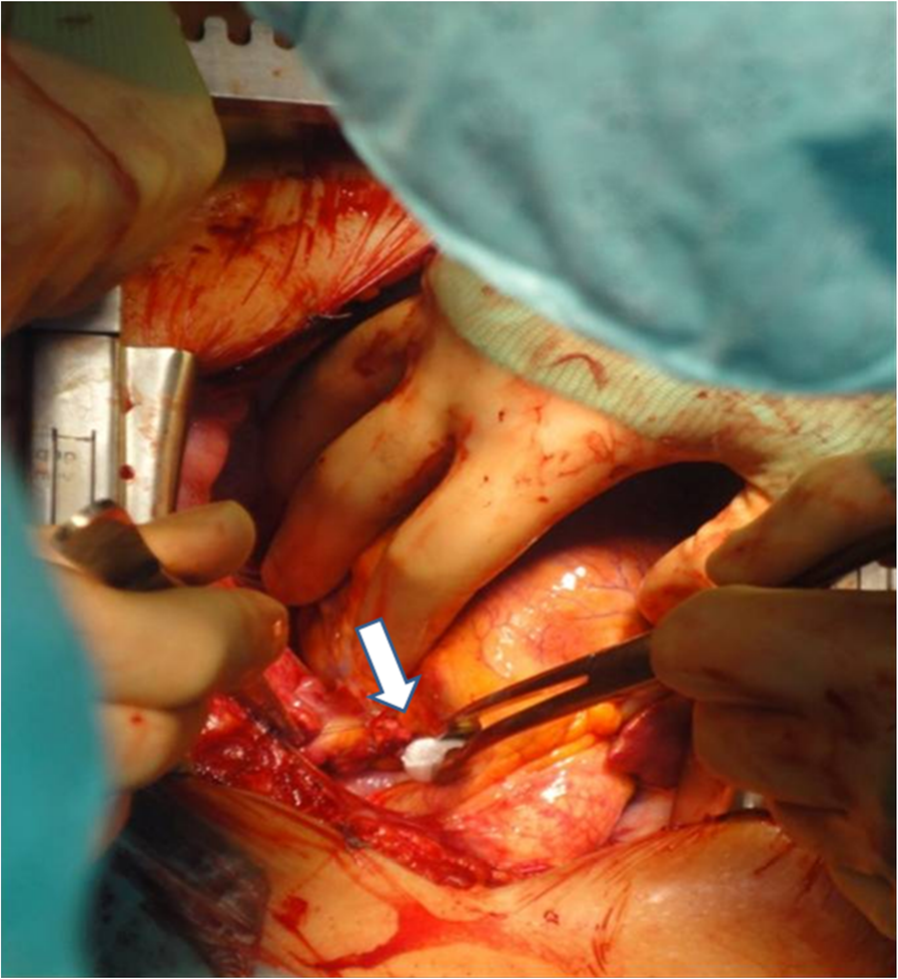
Intraoperative photo of the one-cm left atrial laceration.

Intraoperatively, a combined one-cm laceration of the left atrium and right ventricle was noted (Figure 3). These were repaired with a running suture technique. The patient was successfully extubated seven days after surgery.

## Discussion

Patients with cardiac rupture following blunt thoracic trauma rarely survive and most die at the scene or in the emergency room before the cardiac lesions are disclosed. The most common feature of blunt traumatic cardiac rupture is cardiac tamponade [6]. However, the Beck’s triad (muffled heart sound, engorged jugular vein, and hypotension) has insufficient sensitivity and specificity in cases of multiple systemic trauma. The diagnosis may be delayed because of the co-existing swelling from injury, inadequate hypovolemic correction, or because of the protective cervical collar. If the pericardium has been lacerated, massive hemothorax will be the leading manifestation.

Previous studies showed that blunt cardiac rupture is an exceedingly rare injury, occurring in one of 2400 patients with blunt trauma [7]. The incidence of patients with cardiac rupture reaching the hospital ranges from 0.16% to 2% [1,6]. At autopsy, damage to the heart was found to be the cause of death in 5% to 10% of victims of blunt chest trauma [4,8] with chamber rupture present in 36% to 65% of cases [9].

In patients who arrive alive at the hospital, traumatic blunt cardiac rupture is associated with a high mortality rate [7]; these patients can be saved by prompt diagnosis and immediate, adequate surgical repair [10]. The mortality rate for these patients is between 27.3% and 89% [1,7].

Automobile crashes are the main cause of blunt injury to the heart (73%), followed by pedestrian struck by auto (16%), and falls from a height (8%) [7]. The spectrum of injury varies, including cardiac contusion, rupture of the pericardium or the myocardium (free wall or septum), cardiac valvular disruption, and lacerations of the coronary arteries [4].

According to Santavirta and Arajarvi, more than half of victims of blunt cardiac rupture have six or more fractured ribs or a sternum fracture, indicating a common thoracic crushing force mechanism [11]. Several precordial impactions with cardiac squeezing between the sternum and spine is one possible mechanism [3,4]. In the English literature, however, this is the first case of a patient with pectus excavatum having a blunt rupture. Rationally, the incidence should be higher in these patients because this congenital deformity is a posterior depression of the sternum and adjacent costal cartilages which often compresses the right atrium and ventricle. Another popular theory about blunt traumatic cardiac rupture is rapid decelerations with resultant disruption of the atria from their connections to the vena cava and pulmonary veins [6].

Seat belt use has been mandatory in Germany since 1976 for front seat passengers, and since 1990 for the rear seat. Due to the increase in road traffic accidents and seat belt compliance, the rate of injuries resulting from seat belt use are on the rise. A specific pattern of injuries such as sternal fracture, bowel trauma, or lumbar spine injuries have been categorized as part of the seat belt syndrome [12]; the sternal fracture is the most common seat belt injury. The incidence of traumatic cardiac rupture remains remarkable in fatally injured seatbelt wearers [11].

## Conclusion

We have reported the case of a patient who was brought to our hospital because of a complaint of thoracic pain. She probably would not have survived if brought to a hospital without a cardiothoracic surgery department. Because the most common mechanism of blunt cardiac rupture is squeezing of the heart between the sternum and the spine, we believe that patients with pectus excavatum are at a higher risk for heart ruptures and suggest their admission to Level I Trauma Centers.

## Consent

Written informed consent was obtained from the patient for publication of this case report and accompanying images. A copy of the written consent is available for review by the Editor-in-Chief of this journal.

## Competing interests

The authors declare that they have no competing interests.

## Authors’ contributions

EL and EL analyzed and interpreted the patient data and searched the existing literature on the topic. MP and NH found the photos and designed the figures. MJ and CK came up with the idea of writing the case report and corrected the manuscript. HB assisted with the writing and editing of the manuscript. All authors read and approved the final manuscript.

## Supplementary Material

Additional file 1 **Table S1. **Blood gas analysis of the patient on admission and after 45 minutes. Click here for file
