# Supplementary material for: Pectus excavatum in blunt chest trauma: a case report
Source: J Med Case Rep. 2013 Jan 15;7:22. doi: 10.1186/1752-1947-7-22 (PMC3567954; doi:10.1186/1752-1947-7-22)
Supplement: Additional file 1 — Table S1. Blood gas analysis of the patient on admission and after 45 minutes. [file 1752-1947-7-22-S1.docx]

**Additional file 1:** **Table S1. Blood gas analysis of the patient on admission and after 45 minutes.**

| **Blood gas analysis’ parameters** | **On admission** | **45min after admission** |
| --- | --- | --- |
| **Hb (g/dl)** | 10.8 | 9.3 |
| **Blood pressure (mmHg)** | 120/70 | 70/40 |
| **Heart frequency(pulses/min)** | 84 | 122 |
| **pH** | 7.35 | 7.26 |
| **Lactate(mmol/l)** | 1.1 | 8.6 |
| **SO2 (%)** | 94 | 99 (6l O2) |

Hb, hemoglobin
